# Wetting Properties of a Saponin-Rich Aqueous Soapwort Extract

**DOI:** 10.3390/molecules30163413

**Published:** 2025-08-18

**Authors:** Anna Zdziennicka, Katarzyna Szymczyk, Bronisław Jańczuk, Kamil Wojciechowski, Ewa Kobylska

**Affiliations:** 1 Department of Interfacial Phenomena, Institute of Chemical Sciences, Faculty of Chemistry, Maria Curie-Skłodowska University in Lublin, Maria Curie-Skłodowska Sq. 3, 20-031 Lublin, Poland; katarzyna.szymczyk@mail.umcs.pl (K.S.); bronislaw.janczuk@mail.umcs.pl (B.J.); 2 Faculty of Chemistry, Warsaw University of Technology, Noakowskiego 3, 00-664 Warsaw, Poland; kamil.wojciechowski@pw.edu.pl (K.W.); ewa.kobylska@pw.edu.pl (E.K.); 3 Department of Chemistry, University of Warmia and Mazury in Olsztyn, Pl. Łódzki 4, 10-721 Olsztyn, Poland

**Keywords:** saponin extract, wettability, surface tension

## Abstract

The saponin-rich plant extracts are mixtures of various surface-active and non-surface-active compound substances. Their exact composition depends on the type of plant and its part from which they were extracted. In this study, we analyze the wetting properties of the extract obtained by boiling soapwort (*Saponaria officinalis* L.) roots in water (SE). To this aim, the contact angle measurements of aqueous solutions of SE on apolar (AP) (polytetrafluoroethylene, PTFE), monopolar (MP) (polymethyl methacrylate, PMMA), weak bipolar (WBP) (composites with varying content of cellulose and chitosan), and bipolar solids (BP) (quartz) were determined. The surface tension of the solids used for the contact angle measurements ranged from 20.24 to 47.7 mN/m. Based on the measured contact angles, the relationship between adhesion and surface tension, the cosine of the contact angle and surface tension, the cosine of the contact angle and the reciprocal of the surface tension, as well as the adsorption of the surface-active components of SE at the solid-solution and solid-air interfaces were analyzed. The results indicate that the adsorption of SE components at the hydrophobic solid-solution interface is comparable to that at the solution–air interface. Moreover, the Gibbs free energy of adsorption at the solid-air interface for all solids studied is comparable to that at the solution–air interface.

## 1. Introduction

In recent years, there has been an increase in interest in the physicochemical properties of saponin-rich plant extracts. This is due, among other reasons, to their increasingly common use in practice [1,2,3,4,5,6,7,8,9,10,11,12,13,14,15]. The use of saponins, among other applications, as additives to soaps and shower gels as well as in plant protection products in ecological cleaning agents [16,17,18,19] is closely related to the wetting and adhesive properties of their aqueous solutions [20,21]. These properties can be deduced based on the contact angle of saponin aqueous solutions on model apolar, monopolar, and bipolar solids. The values of the contact angle and adhesion work of the aqueous solution to the solid surface depend on the surface tension of the solution and solid, as well as the solid-solution interfacial tension [22]. A contact angle equal to zero indicates that complete spreading of the solution over the solid surface takes place. In such cases, the adhesion work of the solution is equal to the cohesion work of the solution. Thus, measurements of the contact angle of the aqueous solution of the saponin-rich soapwort extract on model solids (θ) allow us to establish a criterion for the complete spreading of the liquid or solution over the solid surface [22]. One method for establishing this criterion is based on the dependence of the cosine of the contact angle on the liquid surface tension ($\gamma_{LV}$) [23,24,25,26], and another on the relationship between adhesion ($\gamma_{LV}cos\theta$) and surface tension [27]. By extrapolating these relationships to the value of surface tension corresponding to the zero contact angle, the value of the so-called critical surface tension of solid wetting ($\gamma_{C}$) can be determined. This is possible if the above-mentioned dependences can be described by an appropriate mathematical function. Zisman and Bernett [23,24,25,26] suggested that regardless of whether pure liquids or other liquids are employed (including aqueous solutions of surfactants), the relationship between cosθ and $\gamma_{LV}$ is linear. In turn, other authors [28] claim that in most cases there is a linear relationship between $\gamma_{LV}cos\theta$ and $\gamma_{LV}$, but this dependence can have a different slope relative to the X axis for the given solid depending on the type of liquid used for the contact angle measurements. The slope of the linear dependence between adhesion and surface tension obtained based on the contact angle for apolar liquids is different from that obtained from the contact angle of polar liquids and different from those determined from the contact angle of aqueous solutions of surfactants [28]. However, it should be noted that in the case of solutions, for some solids, the relationship between $\gamma_{LV}cos\theta$ and $\gamma_{LV}$ is not strictly linear over the entire concentration range [28,29]. Moreover, the slope of the curve depends on the type of solution. Similar conclusions result from the analysis of the relationship between cosθ and $\gamma_{LV}$ [28,29]. Therefore, based on the isotherms expressing the dependence of adhesion and the cosine of the contact angle on surface tension, it can be concluded that the total wetting of a liquid or solution on the surface of a given solid depends not only on the value of the surface tension of the liquid or solution but also on its components and parameters. The possibility of forming an adsorption layer of the solution components on the surface of a solid beyond the deposited drop, which affects the value of the contact angle, may also change the value of the solid surface tension at the zero contact angle [30]. The presented wetting process research methods have been used so far for various pure liquids and aqueous binary or ternary solutions. However, it is difficult to find studies on the wetting and adhesion characteristics of solutions as complex as plant extracts.

The wetting as well as adhesion properties of liquids, particularly solutions, depend more on the properties of adsorbed layers at the solid-air, liquid-air, and solid-liquid interfaces than on those of pure liquids. For this reason, it is difficult to find studies in the literature that deal with the wetting and adhesion properties of multicomponent solutions with regard to the adsorption of solution components at the three interfaces.

Since plant extracts are mixtures of various surface-active and non-surface-active compounds, their wetting properties are little known. Moreover, the composition of the extract depends on the type of plant, the organs from which it was extracted, as well as the method of extraction. Soapwort (*Saponaria officinalis* L.) is one of the richest sources of surface-active saponins, which are distributed in all organs of the plant. However, its roots are by far the most popular source of saponins for various pharmaceutical, cosmetic, and food applications, e.g., in expectorants, shampoos, and halva, to name just a few. The aim of the presented studies was to establish the wetting and adhesion properties of the extract obtained by boiling the soapwort roots in water (SE). This aim was accomplished by measuring the contact angle of aqueous solutions of SE on model solids: apolar (AP) (polytetrafluoroethylene, PTFE), monopolar (MP) (polymethyl methacrylate, PMMA), and bipolar (BP) (quartz). In addition, measurements were performed on weak bipolar (WBP) composites based on bisphenol A diacrylate (BPA.DA) and *N*-vinyl-2-pyrrolidone (NVP) (BPA.DA+NVP) with cellulose (CEL) and chitosan (CHI) as eco-friendly fillers. The tested composites contained 5 to 20% of cellulose (5CEL, 10CEL, 15CEL and 20CEL) and 5 to 15% of chitosan (5CHI, 10CHI, and 15CHI). Earlier studies proved that the addition of CEL and CHI to the acrylate composite (BPA.DA+NVP) increased its thermal resistance and hydrophobicity [31,32]. The solids used for the studies of the wetting and adhesion properties of SE were chosen according to the practical applications of saponins. PTFE and PMMA, for example, are widely used in medicine as surgical and orthopedic biomaterials [33,34]. The results obtained were analyzed as a function of the surface tension of the aqueous solution of SE and the solid, as well as the relationship between the contact angle and SE adsorption at the solid-solution(S-Sol) and solid-air (S-A) interfaces. Additionally, the Gibbs free energy of adsorption of SE at these interfaces was determined and compared with that at the solution–air interface. The adsorption of SE components on the solid-air, solid-liquid, and liquid-air interfaces and the thermodynamic parameters of this adsorption were determined for the first time and discussed within the framework of a model saponin consisting of the selected aglycone (hederagenin) tail and a sucrose head.

## 2. Theory

### 2.1. Relation Between the Contact Angle and the Liquid-Air, Solid-Air, and Solid-Liquid Interface Tension

The contact angle reflects the wetting properties, among others, of the aqueous solutions of surfactants and is related to solid-air, liquid-air, and solid-liquid interface tensions. In turn, these interface tensions are related to the adsorption of surfactants at the solid-air, liquid-air, and solid-air interfaces.

Briefly, 220 years ago, on the basis of interface tensions, Young discussed the equilibrium state of a liquid droplet deposited on a solid surface [35]. Next, Dupre provided a thermodynamic form to these considerations, formulating the so-called Young equation:

(1) $$\gamma_{SV}-\gamma_{SL}=\;\gamma_{LV}cos\theta ,$$

where $\gamma_{SV}$ is the solid surface tension, $\gamma_{LV}$ is the liquid or solution surface tension, $\gamma_{SL}$ is the solid-liquid or solution interface tension, and θ is the contact angle.

By adding $\gamma_{LV}$ to the left and right sides of Equation (1), one obtains

(2) $$\gamma_{SV}+\gamma_{LV}-\gamma_{SL}=\gamma_{LV}\left(cos\theta +1\right)=W_{a},$$

where $W_{a}$ is the adhesion work of the liquid to the solid surface.

The $\gamma_{LV}\left(cos\theta +1\right)=W_{a}$ is known as the Young–Dupre equation [36]. From Equation (2), it follows that

(3) $$\gamma_{LV}cos\theta =-\gamma_{LV}+W_{a},$$

and

(4) $$cos\theta =-1+\frac{W_{a}}{\gamma_{LV}},$$

Equations (3) and (4) are fulfilled if there is a linear dependence between $\gamma_{LV}cos\theta$ and $\gamma_{LV}$ (slope equal to −1), and the linear dependence between cosθ and $\frac{1}{\gamma_{LV}}$ intersects the Y axis at the point equal to −1. In such a case, $W_{a}$ is constant for a given series of liquids, and for aqueous surfactant solutions, it does not depend on their concentration.

Unfortunately, in most cases, $W_{a}$ depends on the concentration of the surfactants, and it is difficult to explain the wetting process. Therefore, to explain this process on the basis of the Young equation, the dependence between solid-liquid interface tension and solid and liquid surface tension must be known. Taking this into account, Neumann et al. [37,38,39] derived the following equation:

(5) $$\frac{cos\theta +1}{2}=\sqrt{\frac{\gamma_{SV}}{\gamma_{LV}}}exp\left[-\beta {\left(\gamma_{LV}-\gamma_{SV}\right)}^{2}\right],$$

where β is the constant equal to 0.000115 (m^2^/mJ)^2^, which according to Neumann et al. does not depend on the kind of solid and liquid.

The analysis of the wettability of solids with different surface tensions by the aqueous solution of surfactants based on the relationships between $\gamma_{LV}cos\theta$ and $\gamma_{LV}$, cosθ and $\gamma_{LV}$ and between cosθ and $\frac{1}{\gamma_{LV}}$ as well as Equation (5) showed that in order to explain their wetting properties, it is necessary to understand the components and parameters of surface tension.

Starting from the Young equation and expressing the solid or liquid surface tension as a sum of the Lifshitz van der Waals (*LW*) and the acid–base component (*AB*), the latter being a function of the electron-donor ($\gamma^{-}$) and electron-acceptor parameters ($\gamma^{+}$) ($\gamma_{SV}^{AB}=2\sqrt{\gamma_{SV}^{+}\gamma_{SV}^{-}}$), Van Oss et al. proved that [40,41,42,43]

(6) $$\gamma_{LV}\left(cos\theta +1\right)=2\left(\sqrt{\gamma_{SV}^{LW}\gamma_{LV}^{LW}}+\sqrt{\gamma_{SV}^{+}\gamma_{LV}^{-}}+\sqrt{\gamma_{SV}^{-}\gamma_{LV}^{+}}\right),$$

where γ is surface tension, and *LV* and *SV* refer to the liquid (solution) and solid, respectively.

Equation (6) is fulfilled for bipolar liquids and bipolar solids. For monopolar and apolar solids, Equation (6) simplifies to Equations (7) and (8), respectively:

(7) $$\gamma_{LV}\left(cos\theta +1\right)=2\left(\sqrt{\gamma_{SV}^{LW}\gamma_{LV}^{LW}}+\sqrt{\gamma_{SV}^{-}\gamma_{LV}^{+}}\right),$$

(8) $$\gamma_{LV}\left(cos\theta +1\right)=2\sqrt{\gamma_{SV}^{LW}\gamma_{LV}^{LW}},$$

Equations (6)–(8) can be employed if the liquid vapors behind the drop settled on the solid surface do not influence the solid surface tension. Van Oss [40,41,42,43] and Fowkes [44,45,46] suggested that this occurs when the liquid surface tension is larger than that of the solid on which the liquid drop has settled. When the solution’s $\gamma_{LV}$ > $\gamma_{SV}$ but the solute surface tension is smaller than that of the solid, Equations (6)–(8) assume the following forms:

(9) $$\gamma_{LV}\left(cos\theta +1\right)+\pi_{e}=2\left(\sqrt{\gamma_{SV}^{LW}\gamma_{LV}^{LW}}+\sqrt{\gamma_{SV}^{+}\gamma_{LV}^{-}}+\sqrt{\gamma_{SV}^{-}\gamma_{LV}^{+}}\right),$$

(10) $$\gamma_{LV}\left(cos\theta +1\right)+\pi_{e}=2\left(\sqrt{\gamma_{SV}^{LW}\gamma_{LV}^{LW}}+\sqrt{\gamma_{SV}^{-}\gamma_{LV}^{+}}\right),$$

(11) $$\gamma_{LV}\left(cos\theta +1\right)+\pi_{e}=2\sqrt{\gamma_{SV}^{LW}\gamma_{LV}^{LW}},$$

where $\pi_{e}$ is the adsorption film pressure.

### 2.2. Adsorption of Surfactants at the Solid-Air and Solid-Liquid Interfaces

The wetting properties of surfactants, including saponins, depend on the amount of their adsorption at the solution-air (Sol-A), solid-air (S-A), and solid-solution (S-Sol) interfaces and the orientation and packing of their molecules in the interface layers. The properties of the latter depend on the components and parameters of the surface tension of the tail and head of the surfactant, and that of the solid. The influence of the adsorption of surfactants at the three interfaces on the wetting properties of their aqueous solutions can be established, among other factors, based on the Lucassen–Reynolds equation, which has the following form [47]:

(12) $$\frac{d\left(\gamma_{LV}cos\theta \right)}{d\gamma_{LV}}=\frac{\Gamma_{SV}-\Gamma_{SL}}{\Gamma_{LV}},$$

where Γ is the Gibbs surface excess concentration of surfactants at the given interface (*SV*—solid-air, *SL*—solid-liquid, and *LV*—liquid-air).

If the relationship between $\gamma_{LV}cos\theta$ and $\gamma_{LV}$ is described by the linear equation in the form

(13) $$\gamma_{LV}cos\theta =k\gamma_{LV}+l,$$

then the constant k in this equation is related to $\frac{\Gamma_{SV}-\Gamma_{SL}}{\Gamma_{LV}}$.

If the surfactant molecules do not adsorb at the S-A interface, then based on the k and knowing $\Gamma_{LV},$ the $\Gamma_{SL}$ values can be calculated from Equation (12). The $\Gamma_{SV}$ and $\Gamma_{SL}$ values can also be calculated by applying the equations

(14) $$\Gamma_{SV}=-\frac{m}{RT}{\left(\frac{\partial \gamma_{SV}}{\partial m}\right)}_{T}=-\frac{1}{2.303RT}{\left(\frac{\partial \gamma_{SV}}{\partial logm}\right)}_{T}$$

and

(15) $$\Gamma_{SL}=-\frac{m}{RT}{\left(\frac{\partial \gamma_{SL}}{\partial m}\right)}_{T}=-\frac{1}{2.303RT}{\left(\frac{\partial \gamma_{SL}}{\partial logm}\right)}_{T}.$$

Knowing the $\Gamma_{SV}$ and $\Gamma_{SL}$ values, it is possible to calculate the fraction of coverage of the solid-air and solid-liquid interface by surfactant molecules using the following equation:

(16) $$X^{S}=N\Gamma A_{0},$$

where N is the value of Avogadro number and $A_{0}$ is the limiting area occupied by one molecule.

### 2.3. The Standard Gibbs Free Energy of Adsorption

The literature reports many methods used to calculate the standard Gibbs free energy of surfactant adsorption ($\Delta G_{ads}^{0}$) at various interfaces. However, for each method, the molar concentration and/or mole fraction of the surfactant must be known. To consider the adsorption of the surface-active components of SE at the S-A interface, a model saponin molecule composed of hederagenin as a tail and sucrose as a head can be employed [48]. Using the model saponin, the following Langmuir equation, modified by de Boer [49], was employed for the thermodynamic considerations of the adsorption of SE components at the S-A and S-Sol interfaces:

(17) $$\frac{A_{0}}{A-A_{0}}exp\frac{A_{0}}{A-A_{0}}=\frac{C}{\omega }exp\left(\frac{-\Delta G_{ads}^{0}}{RT}\right),$$

where $A=\frac{1}{\Gamma N}$ and C is the mole concentration of the model saponin at the same mass concentration as SE and ω is the number of the water moles in dm^3^.

(18) $$\frac{C}{\Gamma }=\frac{C}{\Gamma^{max}}+\frac{a}{\Gamma^{max}}$$

where a is the adsorption constant, which is related to $\Delta G_{ads}^{0}$ by the equation

(19) $$a=\omega exp\left(\frac{G_{ads}^{0}}{RT}\right),$$

The constant a can be als calculated based on the Gu and Zhu equation, which has the form [50,51,52]

(20) $$log\frac{\Gamma }{\left(\Gamma^{max}-\Gamma \right)}=logK+nlogC,$$

where $K=\frac{1}{a}$ is the equilibrium constant of the aggregate formation in the surface layer and n is the average number of surfactant molecules or ions in the surface aggregates.

## 3. Results and Discussion

### 3.1. Wetting Properties of the Soapwort Extract (SE)

**Figure 1:**
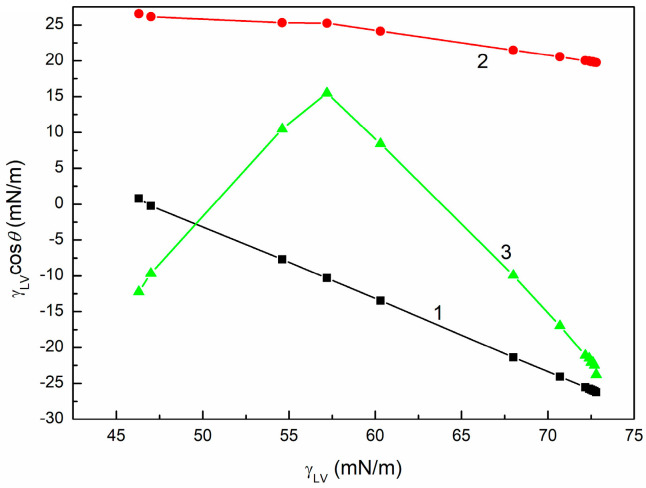
$\gamma_{LV}\cos\theta$ for PTFE (curve 1), PMMA (curve 2), and quartz (curve 3) vs. $\gamma_{LV}$ of SE.

**Figure 2:**
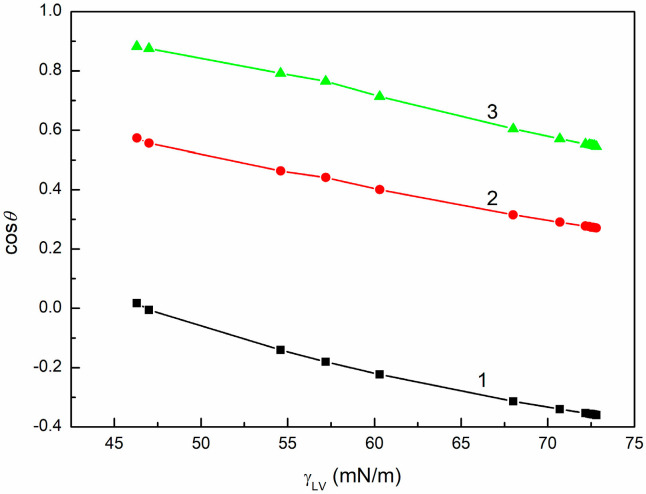
cosθ for PTFE (curve 1), PMMA (curve 2), and quartz (curve 3) vs. $\gamma_{LV}$ of SE.

**Figure 3:**
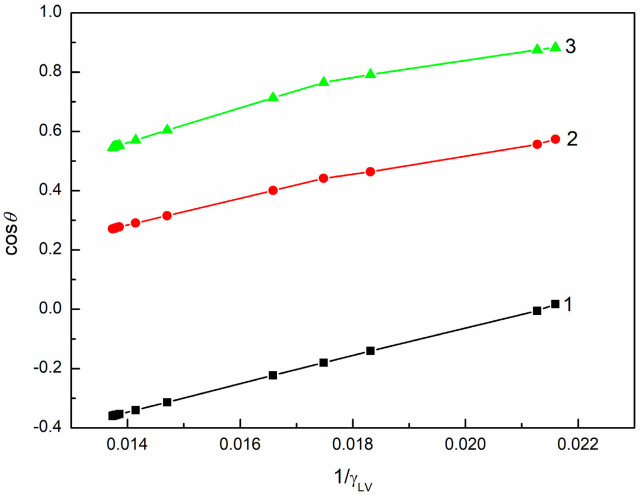
cosθ for PTFE (curve 1), PMMA (curve 2), and quartz (curve 3) vs. $\frac{1}{\gamma_{LV}}$ of SE.

As shown in Figure 1, Figure 2 and Figure 3, the shape of the contact angle isotherms depends on the type of solids. Unfortunately, none of the studied solids exhibited complete spreading of an aqueous solution of SE over their surfaces. Moreover, the minimal values of contact angle on PTFE, PMMA, and quartz surface are higher than those of even synthetic ionic surfactants on these solids [53]. This is probably due to the fact that the surface tension of the saponin tail is higher not only than that of ionic synthetic surfactants but also than the LW component of water surface tension [54].

**Figure 4:**
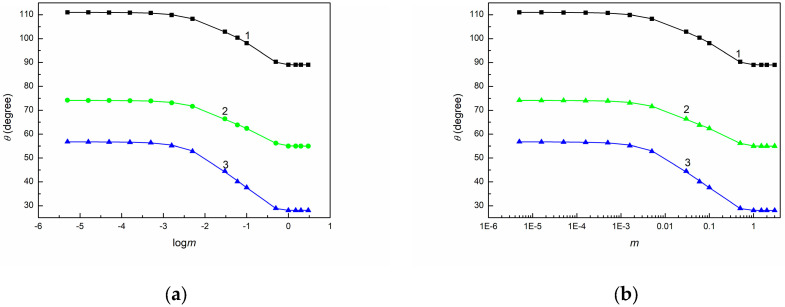
(**a**) θ measured on PTFE (curve 1), PMMA (curve 2), and quartz (curve 3′) vs. logarithm of SE concentration (logm) and (**b**) vs. SE concentration (m).

**Figure 5:**
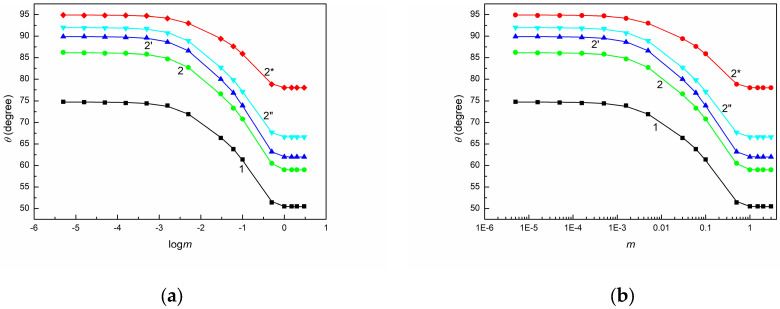
(**a**) θ measured on BPA.DA+NVP (curve 1) as well as 5CEL, 10CEL, 15CEL, and 20CEL (curves 2, 2′, 2”, and 2*) vs. logarithm of SE concentration (logm) and (**b**) vs. SE concentration (m).

**Figure 6:**
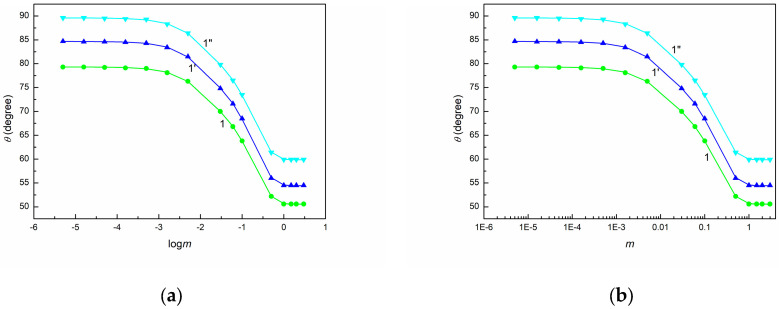
(**a**) θ measured on 5CHI, 10CHI, and 15CHI (curves 1–1”) vs. logarithm of SE concentration (logm) and (**b**) vs. SE concentration (m).

To examine the wetting behavior of SE with regard to the above-mentioned thermodynamic considerations, the dependences between $\gamma_{LV}cos\theta$ and $\gamma_{LV}$ (Figure 1, Figures S1a and S2a), between cosθ and $\gamma_{LV}$ (Figure 2, Figures S1b and S2b), as well as between cosθ and $\frac{1}{\gamma_{LV}}$ (Figure 3, Figures S1c and S2c) were plotted. These dependences were obtained based on the measured contact angles (Figure 4, Figure 5 and Figure 6) and the literature data on the surface tension of aqueous solutions of SE [48].

The dependences between $\gamma_{LV}cos\theta$ and $\gamma_{LV}$ and those between cosθ and $\frac{1}{\gamma_{LV}}$ are linear only for PTFE in the concentration range studied (Figure 1, Figure 2 and Figure 3). However, it should be mentioned that in the case of quartz, two straight relationships between $\gamma_{LV}cos\theta$ and $\gamma_{LV}$ and two different values of $\gamma_{C}$ were obtained (Table 1). Consequently, Equations (3) and (4) are fulfilled (Table 1) only for this solid surface. The statistical analysis of the results presented in Table 1 is provided in Table S1.

Nevertheless, even for PTFE, the $\gamma_{C}$ values determined from the dependence between $\gamma_{LV}cos\theta$ or cosθ and $\gamma_{LV}$ are not equal to $\gamma_{SV}$ (Table 1 and Table 2). Van Oss et al. [40,41,42,43] suggested that the difference between the $\gamma_{C}$ and $\gamma_{SV}$ values may result from a too long extrapolation of $\gamma_{LV}cos\theta$ or cosθ in the range of contact angle values from its minimal measured values to θ = 0. On the other hand, Kitazaki and Hata et al. [55,56,57] suggested that in the case of apolar solids, the condition $\gamma_{C}$ = $\gamma_{SV}$ is fulfilled only for apolar liquids. The $\gamma_{SV}$ value of apolar PTFE was determined based on θ for *n*-alkanes from *n*-heptane (θ close to 0) to *n*-hexadecane [54]. In order to verify the suggestion of Kitazaki and Hata et al. [55,56,57] in the present studies, the dependence between cosθ on $\gamma_{LV}$ was plotted based on the θ values of the contact angle on PTFE for the following liquids: diiodomethane, α-bromonaphthalene, α-chloronaphthalene, 1,2,3-tribromopropane, bromo-benzene, bromoform, and carbon disulfide (Figure S3), whose surface tension is larger than that of *n*-hexadecane (between 32.20 and 50.8 mN/m [54]). The $\gamma_{C}$ value for PTFE determined in such a way (21.01 mN/m) is insignificantly larger than the PTFE surface tension (20.24 mN/m [53]) and close to the surface tension of 20CEL, which is equal to 21.54 mN/m [31]. However, it should be mentioned that the largest contact angle of apolar liquids on the PTFE surface was obtained for diiodomethane, whose surface tension is equal to 50.8 mN/m [54]. This contact angle (74.7°) is smaller than the lowest contact angle measured for SE (Figure 4). This suggests that probably a linear dependence between $\gamma_{LV}cos\theta$ and $\gamma_{LV}$ exists in the studied range of surface tension values. Moreover, $\gamma_{C}$ is not determined solely by the value of the liquid or the solution surface tension, but rather by the components and parameters of the liquids and solids’ surface tension.

**Figure 7:**
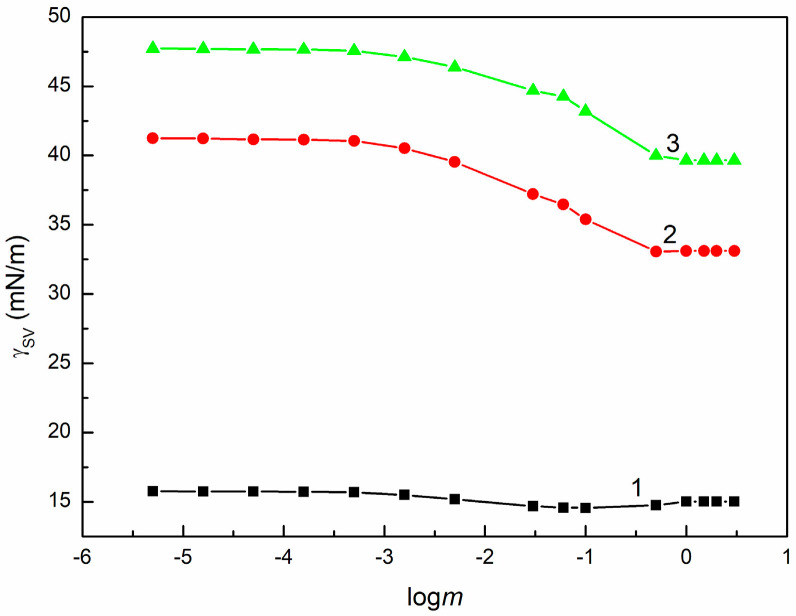
$\gamma_{SV}$ calculated from Equation (5) for PTFE (curve 1), PMMA (curve 2), and quartz (curve 3) vs. logm of SE.

It should also be emphasized that the θ value of SE can be affected by the adsorption layer formed around the solution drop settled on the solid surface due to the transition of some components of SE from the drop to the solid surface [30]. This is confirmed by the calculations of solids’ surface tension from θ of SE measured on the surface of the studied solids using the Neumann et al. equation [37,38,39]. Apart from PTFE, the $\gamma_{SV}$ values obtained decrease with increasing SE concentration in the aqueous solution (Figure 7, Figures S4 and S5).

It is interesting that for the SE concentrations larger than 1 g/dm^3^, the values of $\gamma_{SV}$ for 20CEL are only 1 mN/m greater than those for PTFE. Also, in the case of the 20CEL plate, the weakest wettability was demonstrated in this range of SE concentrations (the difference between the contact angle for water and SE was only 17°). The contact angle values of the aqueous solutions of SE suggest that PTFE, PMMA, and cellulose-based plates are poor wetting agents, which is in agreement with previous observations for a different plant source and using a different method of extraction [58]. In the case of quartz and chitosan plates, the wettability was the largest.

### 3.2. Components and Parameters of SE Surface Tension and Their Applicability

**Figure 8:**
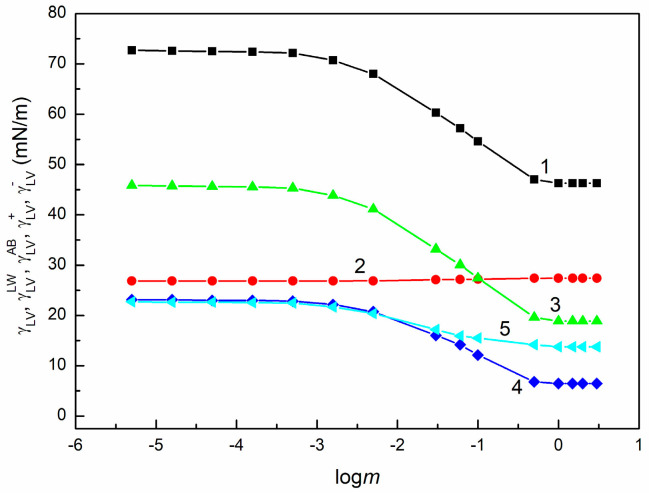
$\gamma_{LV}$ (curve 1), $\gamma_{LV}^{LW}$ (curve 2), $\gamma_{LV}^{AB}$ (curve 3), $\gamma_{LV}^{+}$ (curve 4), and $\gamma_{LV}^{-}$ (curve 5) of the SE vs. logarithm of its concentration (logm).

The calculation of the $\gamma_{SV}$ values for PTFE from Equation (5) based on $\gamma_{LV}$ of SE and θ of this solution on PTFE showed that these values are constant within the error range, independently of the SE concentration (Figure 7). This indicates that the surface tension of saponins and other extracted compounds is smaller than the $\gamma_{SV}$ of PTFE and 20CEL (Table 2) [27,37]. Thus, it was possible to calculate the $\gamma_{LV}^{LW}$ of SE from Equation (8). The obtained values of $\gamma_{LV}^{LW}$ do not depend on the SE concentration and are close to the Lifshitz van der Waals component of water surface tension, which is equal to 26.85 mN/m [48] (Figure 8).

As shown in Figure 8, the adsorption of the surface-active SE components at the S-A interface reduces only the AB component of water, $\gamma_{LV}$. Above the critical micelle concentration (CMC) of SE, $\gamma_{LV}^{AB}$ becomes smaller than $\gamma_{LV}^{LW}$ [48]. However, the minimum value of $\gamma_{LV}^{LW}$ for SE is still higher than that for synthetic nonionic surfactants (Tritons) and other biosurfactants (rhamnolipid and surfactin) [59]. These results are likely due to the fact that the aglycones constituting the tails of saponins present in SE are able to form hydrogen bonds with water molecules [48].

To determine the $\gamma_{LV}^{+}$ values of the AB component of $\gamma_{LV}$ of SE, the contact angle of SE solutions on the PMMA surface were taken into account. The latter surface is abundant in -CO groups possessing electron-donor properties. The lack of any chemical groups with electron-acceptor properties makes PMMA a monopolar (MP) solid. Therefore, for liquids with $\gamma_{LV}$ higher than $\gamma_{SV}$ of PMMA, Equation (7) would be fulfilled. Unfortunately, contrary to PTFE, the surface tension of PMMA calculated from Equation (5) changes as a function of SE concentration, implying $\pi_{e}>0$ for PMMA. Assuming that the $\pi_{e}$ value for a given concentration of SE is equal to the difference between the $\gamma_{SV}$ of PMMA calculated from the water contact angle and that determined from the θ of the solution for this concentration, it was possible to use Equation (10) to determine the $\gamma_{LV}^{+}$ of SE. In turn, the $\gamma_{LV}^{-}$ of SE can be obtained from the expression $\gamma_{SV}^{AB}=2\sqrt{\gamma_{SV}^{+}\gamma_{SV}^{-}}$. It appeared that for the given concentration of the aqueous SE solution, the $\gamma_{LV}^{+}$ value is lower than that of $\gamma_{LV}^{-}$, contrary to what was observed for surfactants without polar groups in their tails. As was determined earlier, for hederagenin, which can be treated as a model tail of a saponin molecule, the component $\gamma_{LV}^{-}$ > 0 and $\gamma_{LV}^{+}$ = 0 [48]. Perhaps for this reason, the $\gamma_{LV}^{-}$ parameter for SE is higher than $\gamma_{LV}^{+}$ (Figure 8).

**Figure 9:**
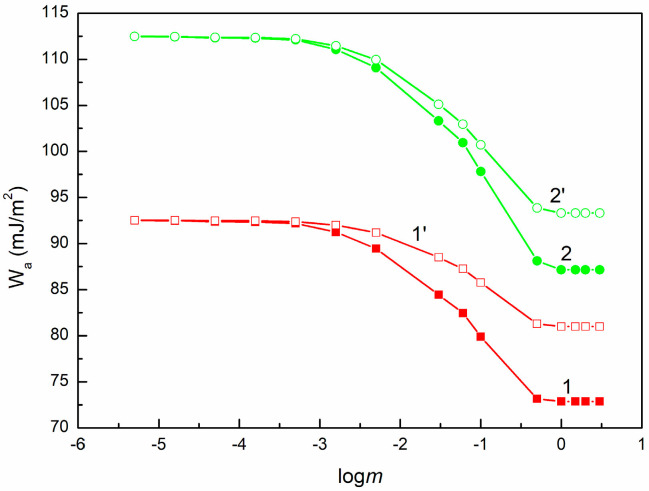
$W_{a}$ for PMMA (curves 1 and 1′) and quartz (curves 2 and 2′) vs. logm of SE. Curves 1 and 2 correspond to the values calculated from Equation (2), and curves 1′ and 2′ to the values calculated from $2\left(\sqrt{\gamma_{SV}^{LW}\gamma_{LV}^{LW}}+\sqrt{\gamma_{SV}^{+}\gamma_{LV}^{-}}+\sqrt{\gamma_{SV}^{-}\gamma_{LV}^{+}}\right)=W_{a}$.

In the next step, we examined the applicability of the components and parameters of surface tension determined from θ on the PTFE and PMMA surfaces to predict the wettability of other solids. To this aim, the adhesion work of the SE solution to PMMA, quartz, as well as the composites with different contents of cellulose and chitosan was determined using the above-mentioned Young–Dupre equation and the van Oss et al. equation [40,41,42,43] ($2\left(\sqrt{\gamma_{SV}^{LW}\gamma_{LV}^{LW}}+\sqrt{\gamma_{SV}^{+}\gamma_{LV}^{-}}+\sqrt{\gamma_{SV}^{-}\gamma_{LV}^{+}}\right)=W_{a}$) (Figure 9, Figures S6 and S7).

For calculations of $W_{a}$, the components and parameters of SE surface tension determined in the present studies were combined with the corresponding literature values for the solids [31,32,59].

Based on the results obtained, it can be stated that θ for SE on a given solid can be well predicted, provided that its surface tension is comparable or smaller than that of hederagenin. In other case, the $W_{a}$ values determined from the Young–Dupre and van Oss et al. equations differ significantly. The surface-active components of SE can adsorb onto surfaces like PMMA, quartz, 5CEL, 10CEL, or 5CHI (Figure 9, Figures S6 and S7), decreasing the solid’s surface tension and biasing the value of adhesion work determined by the two methods.

### 3.3. SE Concentration at the Solid-Air, Solid-Solution, and Solution-Air Interfaces

For almost all systems studied, with the exception of the quartz surface, a linear dependence between $\gamma_{LV}cos\theta$ and $\gamma_{LV}$ was observed in the whole range of the SE concentrations investigated. In the case of quartz, there are two linear dependences between $\gamma_{LV}cos\theta$ and $\gamma_{LV}$, one with a positive slope and the other with a negative one. It should be noted that the positive slope of the linear relationship does not imply a negative adsorption of the SE components at the quartz-solution (quartz-Sol) interface. Instead, this suggests that their adsorption at the quartz-air (quartz-A) interface is larger than at the quartz-Sol interface. However, two different linear relationships between $\gamma_{LV}cos\theta$ and $\gamma_{LV}$ in different concentration ranges can result from the fact that a highly ordered layer of water is formed on the quartz surface, which is difficult for surfactant molecules, such as saponins from SE, to penetrate at low concentrations [60,61,62,63]. Probably for this reason, depending on the SE concentration, its surface-active components (e.g., saponins) adsorb preferentially at the quartz/water layer-solution or quartz-solution interfaces.

Since adsorption of the SE components by penetration from the drop onto the surface of most of the tested solids cannot be ruled out, adsorption at the respective interfaces (solid-solution, $\Gamma_{SL}$, and solid-air, $\Gamma_{SV}$) was calculated using the isotherms Equations (14) and (15).

In Equations (14) and (15), all surface-active components of SE are treated as a single one, and its activity is assumed to be equal to the mole fraction of saponins (the activity coefficient is assumed to be close to unity).

**Figure 10:**
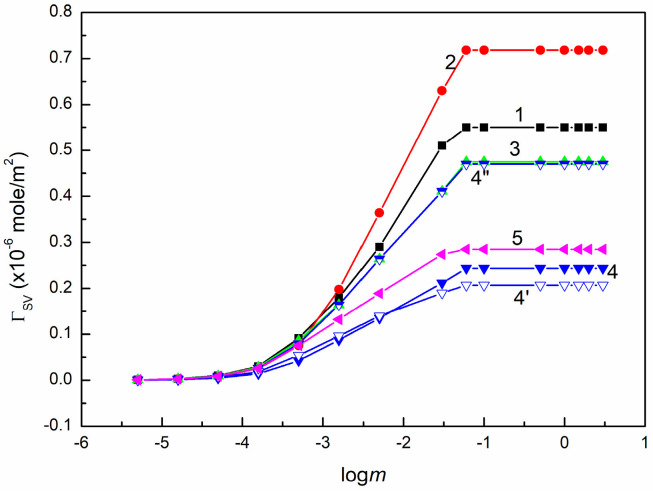
$\Gamma_{SV}$ on PMMA (curve 1), quartz (curve 2), BPA.DA+NVP (curve 3), 5CEL, 15CEL, 20CEL (curves 4, 4′ and 4”), and 5CHI (curve 5) vs. logarithm of SE concentration (logm).

**Figure 11:**
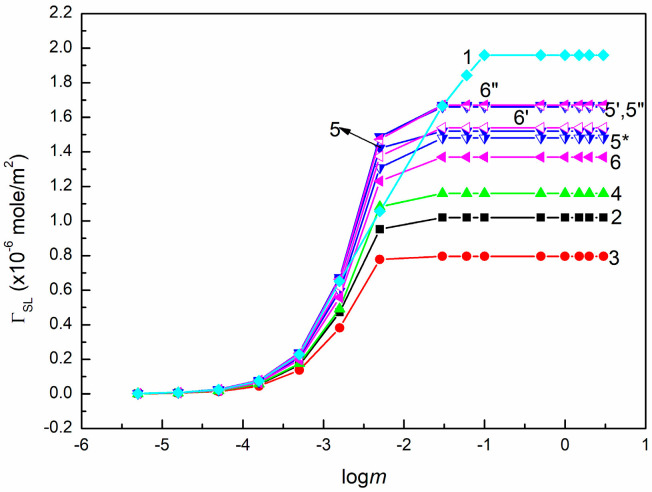
$\Gamma_{SL}$ on PTFE (curve 1), PMMA (curve 2), quartz (curve 3), BPA.DA+NVP (curve 4), 5CEL, 15CEL, 20CEL (curves 5, 5′, 5” and 5*), 5CHI, 10CHI, and 15CHI (curves 6, 6′ and 6”) vs. logarithm of SE concentration (logm).

The $\gamma_{SV}$ value used in Equation (14) for the determination of $\Gamma_{SV}$ was established from the Neumann et al. equation [38,39,40]. In turn, the $\gamma_{SL}$ used in Equation (15) for the $\Gamma_{SL}$ calculation was obtained from Equation (1) based on $\gamma_{SV}$ calculated from the Neumann equation. As follows from the calculations of the Gibbs surface excess concentration of the SE components at the S-A and S-Sol interfaces, for almost all studied solids (with the exception of PTFE), $\Gamma_{SV}<\;\Gamma_{SL}<\;\Gamma_{LV}$ (Figure 10 and Figure 11). For PTFE, $\Gamma_{SV}=0$ and $\Gamma_{SL}$ is insignificantly larger than $\Gamma_{LV}.$

The values of $\Gamma_{SV}$ and $\Gamma_{SL}$ depend on the components and parameters of the surface tension of the solids studied. An increase in these components and parameters increases $\Gamma_{SV}$ and decreases $\Gamma_{SL}$, which likely results from changes in the orientation of the surface-active components of SE (e.g., saponins) in the surface layers, shifting from a more perpendicular to a more parallel arrangement. In such cases, the saturation of the surface layer by saponin molecules takes place at a lower number of molecules. This is confirmed by calculations of the surface covered by saponin molecules ($X^{S}$) from Equation (16) (Figures S8 and S9):

If the same $A_{0}$ (74.4 Å^2^) as at the S-A interface is taken into account, then the calculated values of $X^{S}$, with exception of PTFE, seem to be smaller than real ones. Indeed, it is difficult to assume that the molecules of saponins can be completely oriented in parallel. The $X^{S}$ value depends to a large extent on the mutual orientation of particular fragments of the saponin molecule in the surface layer.

**Figure 12:**
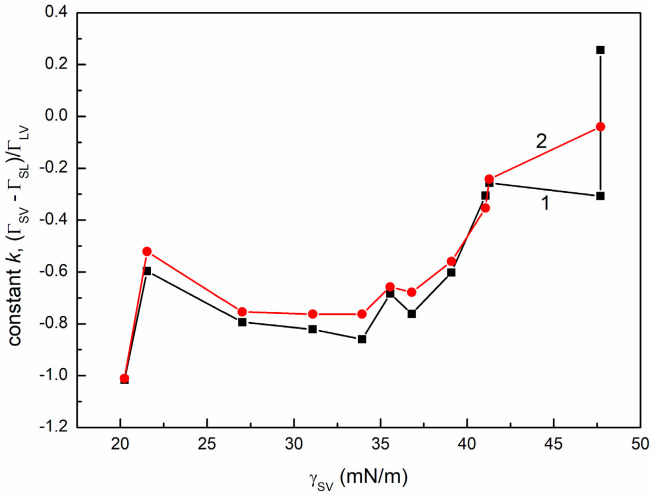
The constant k (curve 1) and the value of the ratio $\left(\Gamma_{SV}-\Gamma_{SL}\right)/\Gamma_{LV}$ (curve 2) vs. $\gamma_{SV}\;$ for all solids studied.

As results in Equation (12), $\frac{\Gamma_{SV}-\Gamma_{SL}}{\Gamma_{LV}}$ should be equal to the slope of the linear dependence between $\gamma_{LV}cos\theta$ and $\gamma_{LV}$. To verify this, the term $\frac{\Gamma_{SV}-\Gamma_{SL}}{\Gamma_{LV}}$ was calculated for the Gibbs surface excess concentrations for the saturated monolayers at the S-A, S-Sol, and S-A interfaces. It appeared that, with the exception of quartz, there is good agreement between $\frac{\Gamma_{SV}-\Gamma_{SL}}{\Gamma_{LV}}$ and k values obtained from the relationship between adhesion and surface tension (Figure 12).

As mentioned above, in the case of quartz, there are two linear dependences between $\gamma_{LV}cos\theta$ and $\gamma_{LV},$ and for this reason, there is no agreement between k and $\frac{\Gamma_{SV}-\Gamma_{SL}}{\Gamma_{LV}}$ because the maximum Gibbs surface excess concentration at the quartz-A and quartz-Sol interfaces does not represent the whole range of SE concentration.

### 3.4. Standard Gibbs Free Energy of SE Component Adsorption

The standard Gibbs free energy of adsorption ($\Delta G_{ads}^{0}$) indicates the tendency to adsorb surface-active compounds at different interfaces. The $\Delta G_{ads}^{0}$ can be determined in many ways (see the Theory section).

The calculations performed in different ways show that $\Delta G_{ads}^{0}$ of the model saponin adsorption at the S-Sol interface does not depend on the type of solid and is close to the adsorption energy at the S-A interface (Figures S10 and S11) [48]. However, $\Delta G_{ads}^{0}$ at the S-A interface depends on the type of solid, and its absolute value is smaller than those for adsorption at the Sol-A and S-Sol interfaces.

It turned out that the adsorption isotherms at both the S-A and S-Sol interfaces satisfy the linear form of the Langmuir adsorption isotherm equation (Equation (18)). The absolute values of $\Delta G_{ads}^{0}$ of the aqueous solutions of SE calculated from Equation (19) are insignificantly larger than those obtained from Equation (17). The results collected in Table 3 confirm that both approaches lead to similar conclusions.

The values of $\Delta G_{ads}^{0}$ obtained from a determined based on Equation (20) are similar to those calculated based on Equation (18) (Table 3). It is also worth emphasizing that the values of a determined from Equations (18) and (20) are similar to those of $\frac{C}{X^{S}}$ (Figures S12 and S13) corresponding to the small concentrations of SE.

## 4. Materials and Methods

### 4.1. Materials

The procedure for obtaining soapwort extract (SE) using only deionized water as a solvent has been described in Ref. [48]. Briefly, the grounded roots of soapwort purchased from Baster-Herbs (Kalisz, Poland) were boiled for 1 h at a plant material/water ratio of 1:10 (*w*/*w*). The extract was cooled down to room temperature and filtered on a Colombo 18 OIL filter press (Rover Pompe, Italy) using 15 µm, 11 µm, 6 µm, and 3 µm paper plates. The filtrate was dried using a YC-015A lab spray dryer (Pilotech, Shanghai, China), with the chamber temperature set to 393 K and the resulting outlet temperature of 343 K. The dried extract was stored at room temperature. The extraction yield (defined as a ratio of the dry mass of the extract to the dry mass of the plant material) was equal to 40.3%, and the total saponin content determined by reversed-phase high-performance liquid chromatography (RP-HPLC) using aescin as a standard amounted to 19.95% (on protoescigenin basis). The SE aqueous solutions were formulated with concentrations varying from 5 × 10^−6^ to 3.0 g/dm^3^. For each SE concentration, three independent solutions were prepared. For two of them, three contact angle measurements were performed, and for one solution, four.

Polytetrafluoroethylene (PTFE) and polymethyl methacrylate (PMMA) were purchased from Mega-Tech (Tomaszow Mazowiecki, Poland) and quartz plates from Conductance (Ostrów Wielkopolski, Poland). The composites based on bisphenol A diacrylate (BPA.DA) and N-vinyl-2-pyrrolidone (NVP) (BPA.DA+NVP) with cellulose (CEL) and chitosan (CHI) were synthesized at Maria Curie-Skłodowska University, as characterized and described earlier [31,32]. All plates were cleaned with soapy water, washed in distilled water many times, and placed in an ultrasonic bath for 20 min. This procedure was repeated twice for each plate. After washing, the plates were dried and placed in a desiccator with molecular sieves. More details have been provided earlier [64].

### 4.2. Methods

Advancing contact angles on the solid surfaces were measured at 293 K, applying the DSA30 measuring system (Krüss, Hamburg, Germany) equipped with a thermostated chamber. For all flat surfaces of PTFE, PMMA, and BPA.DA+NVP as well as plates with CEL and CHI, 10 drops of solution with a volume of 7 µL were used. The liquid volumes satisfy the conditions under which the values of the diameter of the liquid drops—at which the interface pressure of the sphere and hydrostatic pressure at the height of the liquid are equal to the diameter of the drop—are the same [65]. In each experiment, the measuring chamber was saturated with the atmosphere in order to avoid evaporation. The standard deviation of the contact angle values did not exceed ±2°.

## 5. Conclusions

The following conclusions can be drawn from the present results:

The minimum values of the contact angle of aqueous solutions of saponin-rich extract from soapwort (SE) on the apolar, monopolar, weak, and strong bipolar solids suggest that SE is generally a poor wetting agent.

For aqueous solutions of SE, there is a linear relationship between adhesion and surface tension, which allows for the determination of the critical surface tension of PTFE, PMMA, quartz, BPA.DA+NVP, as well as plates with CEL and CHI wetting. However, the slope of the linear relationship between the adhesion and surface tension of aqueous saponin solutions for PTFE is equal to −1. This proves that in the SE concentration range studied, the work of adhesion of their aqueous solutions does not depend on concentration.

Based on the components and parameters of PMMA, quartz, BPA.DA+NVP, plates with CEL and CHI, as well as SE surface tension, it is possible to predict the contact angle isotherms, provided that no surface layer is formed behind the settled solution drop on a given solid, which might decrease the solid surface tension.

The surface tension of the solid covered by the surface-active components of SE can be determined using the Neumann et al. equation.

For PMMA, quartz, BPA.DA+NVP, and plates with CEL and CHI, where a surface adsorption layer is formed beyond the settled SE solution drop, the adsorption of the SE components at the solid-air interface is less than that at the solid-solution and solution–air interfaces.

Surface-active SE components do not adsorb at the PTFE-air interface, and their adsorption at the PTFE-solution interface is comparable to that at the solution–air interface.

Based on the model saponins, whose molecules consist of a hydrophobic aglycone and hydrophilic sucrose, it was possible for the first time to successfully determine the coverage of the solid–aqueous solution of SE and solid-air interfaces by the extract components, to compare this to the coverage at the SE-air interface, as well as to determine the thermodynamic parameters of SE adsorption on these interfaces.

The standard Gibbs free energy of adsorption of SE components at the solid-air and solid-solution interfaces is comparable to this energy at the solution–air interface.

## Figures and Tables

**Table 1:** The values of critical surface tension of solid wetting ($\gamma_{C}$).

Solid	$\gamma_{C}$ [mN/m]	
	$\mathrm{From}\;cos\theta =f(\gamma_{LV})$	$\mathrm{From}\;\gamma_{LV}cos\theta =f(\gamma_{LV})$
PTFE	23.01	23.70
PMMA	27.10	30.80
Quartz	37.35	46.57
		38.46
BPA.DA+NVP	34.61	34.23
5CEL	33.10	33.24
10CEL	32.97	32.85
15CEL	30.80	30.65
20CEL	25.72	23.20
5CHI	36.58	35.67
10CHI	36.44	35.20
15CHI	35.12	33.80

**Table 2:** The components ($\gamma_{SV}^{LW}$, $\gamma_{SV}^{AB}$) and parameters ($\gamma_{SV}^{+}$, $\gamma_{SV}^{-}$) of the solid surface tension ($\gamma_{SV}$).

Solid	$\gamma_{SV}^{LW}$ [mN/m]	$\gamma_{SV}^{AB}$ [mN/m]	$\gamma_{SV}^{+}$ [mN/m]	$\gamma_{SV}^{-}$ [mN/m]	$\gamma_{SV}$ [mN/m]
PTFE	20.24	0.00	0.00	0.00	20.24
PMMA	41.28	0.00	0.00	7.28	41.28
Quartz	39.07	9.63	1.61	14.36	47.70
Hederagenin	29.38	0.00	0.00	6.34	29.38
Saccharose	39.10	3.54	0.1450	21.55	42.63
Saponins head	38.33	3.63	0.1910	20.51	42.29
BPA.DA+NVP	38.4912	2.5695	0.3078	5.3625	41.0607
5CEL	34.8634	0.6922	2.1734	0.0551	35.5556
10CEL	31.0466	0.0319	2.4345	0.0001	31.0785
15CEL	26.8489	0.1644	2.7946	0.0024	27.0133
20CEL	21.2119	0.3231	0.0075	3.4847	21.5350
5CHI	37.1495	1.9447	0.2682	3.5253	39.0942
10CHI	35.3437	1.4580	0.3168	1.6776	36.8017
15CHI	32.9765	0.9502	0.2858	0.7896	33.9267

**Table 3:** The standard Gibbs free energy of SE adsorption ($∆G_{ads}^{0}$).

Solid	$∆G_{ads}^{0}$ (kJ/mol)					
	Solid-Air Interface (S-A)			Solid-Liquid Interface (S-Sol)		
	Equation (17)	Equation (18)	Equation (20)	Equation (17)	Equation (18)	Equation (20)
PTFE	-	-	-	−39.22 ± 0.37	−40.00 ± 0.38	−39.29 ± 0.42
PMMA	−37.00 ± 0.38	−41.05 ± 0.39	−39.92 ± 0.42	−38.52 ± 0.38	−41.98 ± 0.40	−43.18 ± 0.43
Quartz	−36.56 ± 0.37	−40.10 ± 0.40	−39.37 ± 0.43	−38.04 ± 0.37	−42.17 ± 0.40	−43.38 ± 0.42
BPA.DA+NVP	−36.86 ± 0.37	−40.89 ± 0.38	−41.73 ± 0.41	−38.64 ± 0.37	−41.76 ± 0.39	−42.52 ± 0.41
5CEL	−35.12 ± 0.39	−40.98 ± 0.39	−41.68 ± 0.42	−39.42 ± 0.38	−41.84 ± 0.38	−42.65 ± 0.44
10CEL	-	-	-	−39.45 ± 0.39	−41.61 ± 0.39	−42.24 ± 0.42
15CEL	−35.85 ± 0.38	−42.18 ± 0.41	−43.23 ± 0.43	−39.75 ± 0.37	−41.58 ± 0.39	−42.18 ± 0.42
20CEL	−36.85 ± 0.37	−40.96 ± 0.39	−41.51 ± 0.43	−39.38 ± 0.38	−41.57 ± 0.41	−42.16 ± 0.41
5CHI	−36.66 ± 0.38	−42.42 ± 0.40	−43.29 ± 0.42	−39.29 ± 0.39	−41.62 ± 0.40	−42.26 ± 0.42
10CHI	-	-	-	−39.50 ± 0.38	−41.58 ± 0.40	−42.19 ± 0.43
20CHI	-	-	-	−39.70 ± 0.39	−41.53 ± 0.41	−42.10 ± 0.43

## Data Availability

The original contributions presented in the study are included in the article; further inquiries can be directed to the corresponding author.

## Supplementary Materials

The following supporting information can be downloaded at https://www.mdpi.com/article/10.3390/molecules30163413/s1: Table S1. The values of critical surface tension of solid wetting ($\gamma_{C}$)—statistical analysis. Figure S1a. $\gamma_{LV}\cos\theta$ for BPA.DA+NVP, 5CEL, 10CEL, 15CEL, and 20CEL vs. $\gamma_{LV}$. Figure S1b. cosθ for BPA.DA+NVP, 5CEL, 10CEL, 15CEL, and 20CEL vs. $\frac{1}{\gamma_{LV}}$. Figure S1c. cosθ for BPA.DA+NVP, 5CEL, 10CEL, 15CEL, and 20CEL vs. $\frac{1}{\gamma_{LV}}$. Figure S2a. $\gamma_{LV}\cos\theta$ 5CHI, 10CHI, and 15CHI vs. $\gamma_{LV}$. Figure S2b. cosθ for 5CHI, 10CHI, and 15CHI vs. $\gamma_{LV}.$ Figure S2c. cosθ for 5CHI, 10CHI, and 15CHI vs. $\gamma_{LV}$. Figure S3. $\gamma_{LV}\cos\theta$ for PTFE for apolar liquids vs. their $\gamma_{LV}$. Figure S4. $\gamma_{SV}$ calculated from Equation (5) BPA.DA+NVP, 5CEL, 10CEL, 15CEL, and 20CEL vs. logm of SE. Figure S5. $\gamma_{SV}$ calculated from Equation (5) 5CHI, 10CHI, and 15CHI vs. logm of SE. Figure S6. $W_{a}$ for BPA.DA+NVP, 5CEL, 10CEL, 15CEL, and 20CEL vs. logm of SE. Figure S7. $W_{a}$ for 5CHI, 10CHI, and 15CHI vs. logm of SE. Figure S8. $X_{S}$ at the solid-air interface calculated from Equation (16) for PMMA, quartz, BPA.DA+NVP, 5CEL, 15CEL, 20CEL, and 5CHI vs. logm of SE. Figure S9. $X_{S}$ at the solid-solution interface calculated from Equation (16) for PTFE, PMMA, quartz, BPA.DA+NVP, 5CEL, 15CEL, 20CEL, 5CHI, 10CHI, and 15CHI vs. logm of SE. Figure S10. $∆G_{ads}^{0}$ at the solid-air interface calculated from Equation (17) for PMMA, quartz, BPA.DA+NVP, 5CEL, 15CEL, 20CEL, and 5CHI vs. logm of SE. Figure S11 $∆G_{ads}^{0}$ at the solid-liquid interface calculated from Equation (17) for PTFE, PMMA, quartz, BPA.DA+NVP, 5CEL, 15CEL, 20CEL, 5CHI, 10CHI, and 15CHI vs. logm of SE. Figure S12. $\frac{C}{X_{S}}$ at the solid-air interface for PMMA, quartz, BPA.DA+NVP, 5CEL, 15CEL, 20CEL, and 5CHI vs. logm of SE. Figure S13. $\frac{C}{X_{S}}$ at the solid-liquid interface for PTFE, PMMA, quartz, BPA.DA+NVP, 5CEL, 15CEL, 20CEL, 5CHI, 10CHI, and 15CHI vs. logm of SE.
