# Supplementary material for: Wetting Properties of a Saponin-Rich Aqueous Soapwort Extract
Source: Molecules. 2025 Aug 18;30(16):3413. doi: 10.3390/molecules30163413 (PMC12388199; doi:10.3390/molecules30163413)
Supplement: Supplementary file 1 [file molecules-30-03413-s001.zip › molecules-3784200-supplementary.pdf]

Supplementary Information

Wetting Properties of a Saponin-rich Aqueous Soapwort Extract

Anna Zdziennicka [1,*], Katarzyna Szymczyk [1], Bronisław Jańczuk [1], Kamil Wojciechowski [2,3] and Ewa Kobylska [2]

[1] Department of Interfacial Phenomena, Institute of Chemical Sciences, Faculty of Chemistry, Maria Curie-Skłodowska University in Lublin, Maria Curie-Skłodowska Sq. 3, 20-031 Lublin, Poland;
anna.zdziennicka@mail.umcs.pl (A.Z.); katarzyna.szymczyk@mail.umcs.pl (K.S.); bronislaw.janczuk@mail.umcs.pl (B.J.);
[2] Faculty of Chemistry, Warsaw University of Technology, Noakowskiego 3, 00-664 Warsaw, Poland; kamil.wojciechowski@pw.edu.pl (K.W.), ewa.kobylska@pw.edu.pl (E.K.)

[3] Department of Chemistry, University of Warmia and Mazury in Olsztyn, Pl. Łódzki 4, 10-721 Olsztyn, Poland; kamil.wojciechowski@pw.edu.pl (K.W.)

Table S1. The values of critical surface tension of solid wetting (γ_C) – statistical analysis. **(a) –** the γ_C values obtained from $\cos\theta = f(\gamma_{LV})$, (b) – the γ_C values obtained from from $\gamma_{LV}\cos\theta = f(\gamma_{LV})$.

**Solid**	γ_C [mN/m]	**Exponential function**		**Linear function**			
		R2	Chi2/DoF	R	SD	N	P
PTFE	23.01 (a)	0.9992	0.00003			16	
	23.70 (b)			-0.9999765	0.0844037	16	<0.0001
PMMA	27.10 (a)	0.99736	0.00005				
	30.80 (b)			-0.9871175	0.5040711	16	<0.0001
Quartz	37.35 (a)			-0.9991038	0.0065857	16	<0.0001
	46.57 (b)			-0.9993071	0.0582689	10	<0.0001
	38.46 (b)			0.9988726	0.0660785	7	<0.0001
BPA.DA+NVP	34.61 (a)	0.9992	0.00003				
	34.23 (b)			-0.9997054	0.1155373	16	<0.0001
5CEL	33.10 (a)	0.99898	= 0.00005				
	33.24 (b)			-0.9997054	0.1676614	16	<0.0001
10CEL	32.97 (a)	0.99916	0.00004				
	32.85 (b)			-0.9999532	0.0962502	16	<0.0001
15CEL	30.80 9 (a)	0.99904	0.00004				
	30.65 (b)			-0.9998694	0.1554654	16	<0.0001
20CEL	25.72 (a)	0.99957	8.3721E-6				
	23.20 (b)			-0.9992747	0.2754305	16	<0.0001
5CHI	36.58 (a)	0.99944	0.00003				
	35.67 (b)			-0.9998526	0.1252536	16	<0.0001
10CHI	36.44 (a)	0.99941	0.00003				
	35.20 (b)			-0.9997679	0.1990496	16	<0.0001
15CHI	35.12 (a)	0.99943	0.00003				
	33.80 (b)			-0.9998051	0.2057496	16	<0.0001

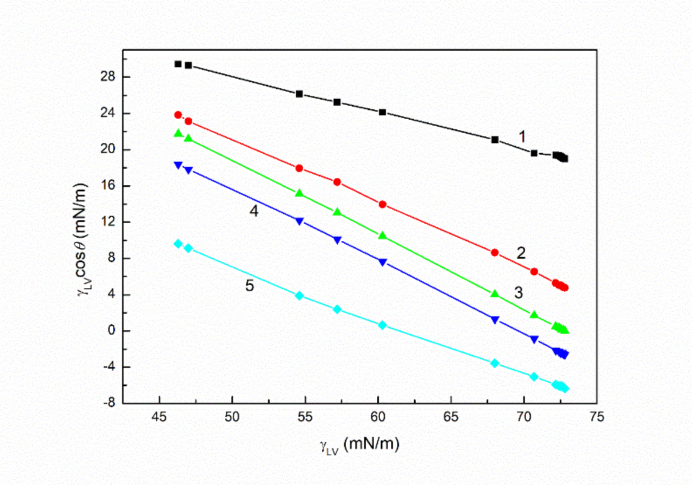

Figure S1a. $\gamma_{LV} \cos\theta$ for BPA.DA+NVP, 5CEL, 10CEL, 15CEL, 20CEL (curves 1 – 5) vs γ_{LV}.

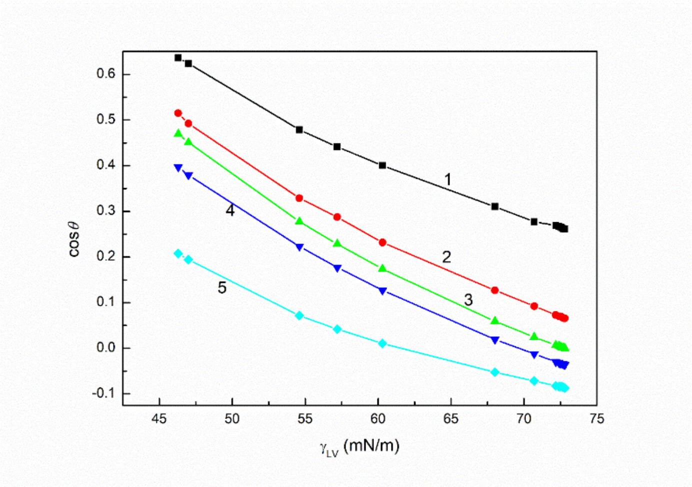

Figure S1b. $\cos\theta$ for BPA.DA+NVP, 5CEL, 10CEL, 15CEL, 20CEL (curves 1 – 5) vs γ_{LV}.

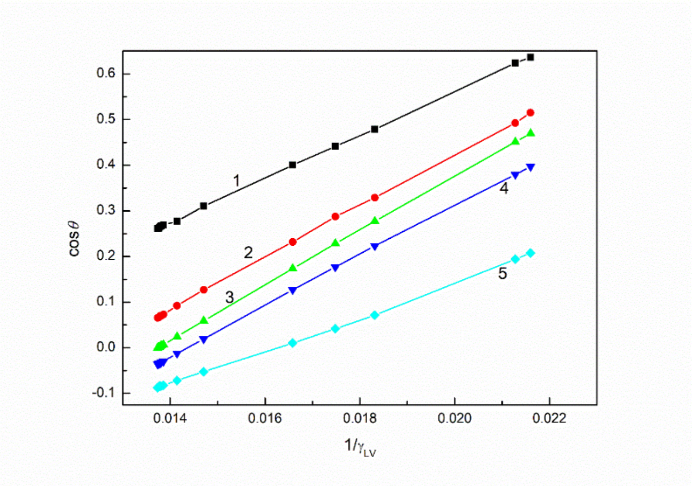

Figure S1c. $\cos\theta$ for BPA.DA+NVP, 5CEL, 10CEL, 15CEL, 20CEL (curves 1 – 5) vs $\frac{1}{\gamma_{LV}}$.

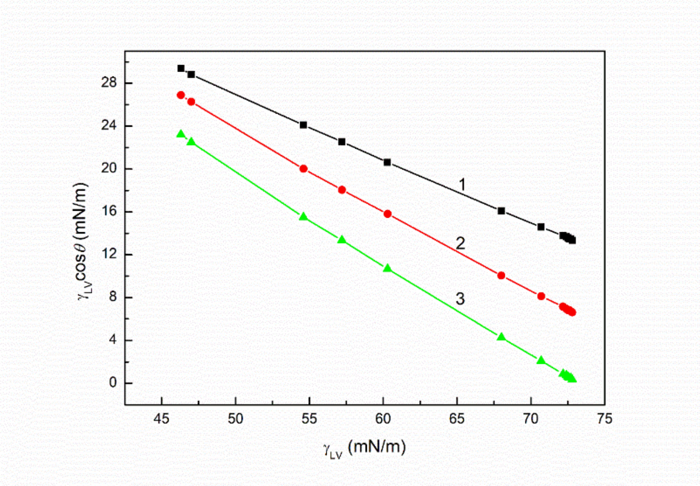

Figure S2a. $\gamma_{LV}\cos\theta$ 5CHI, 10CHI and 15CHI (curves 1 – 3) vs γ_{LV}.

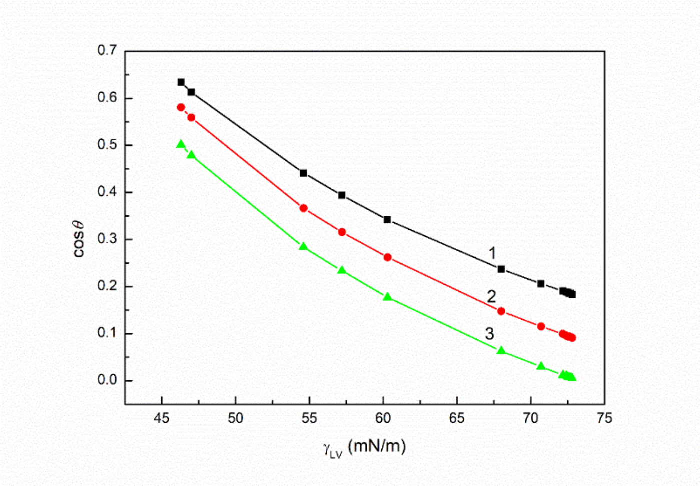

Figure S2b. $\cos\theta$ for 5CHI, 10CHI and 15CHI (curves 1 – 3) vs γ_{LV}.

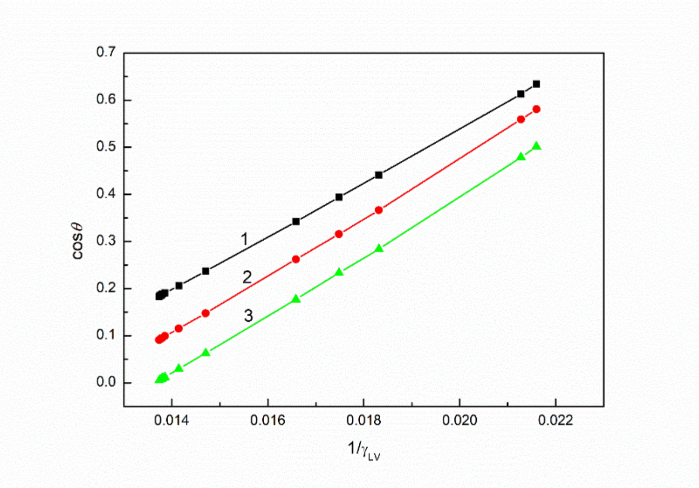

Figure S2c. $\cos\theta$ for 5CHI, 10CHI and 15CHI (curves 1 – 3) vs $\frac{1}{\gamma_{LV}}$.

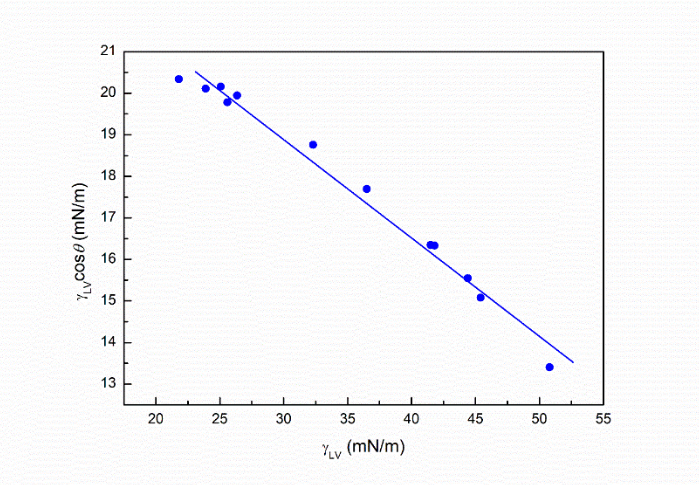

Figure S3. $\gamma_{LV}\cos\theta$ for PTFE for apolar liquids vs their γ_{LV}.

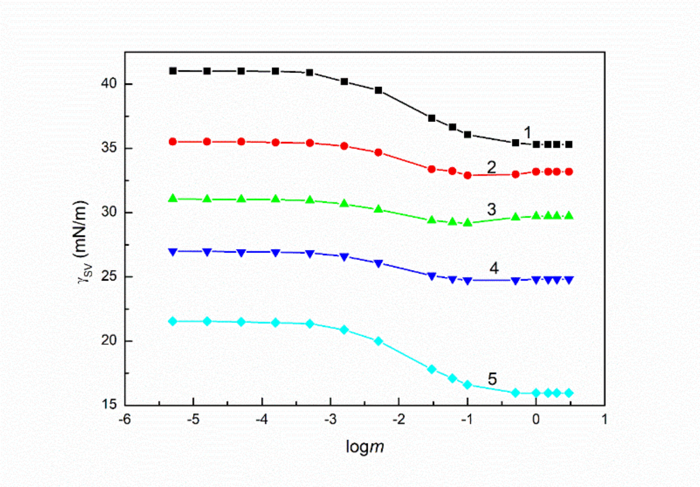

Figure S4. γ_{SV} calculated from Eq. (5) BPA.DA+NVP, 5CEL, 10CEL, 15CEL, 20CEL (curves 1 – 5) vs log*m* of SE.

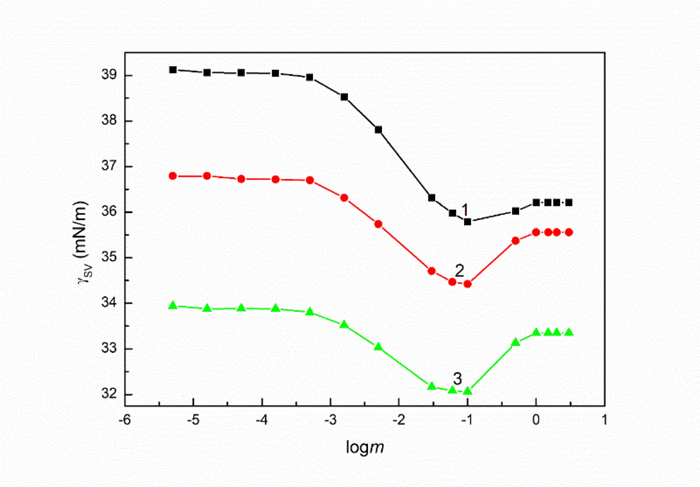

Figure S5. γ_{SV} calculated from Eq. (5) 5CHI, 10CHI and 15CHI (curves 1 – 3) vs log*m* of SE.

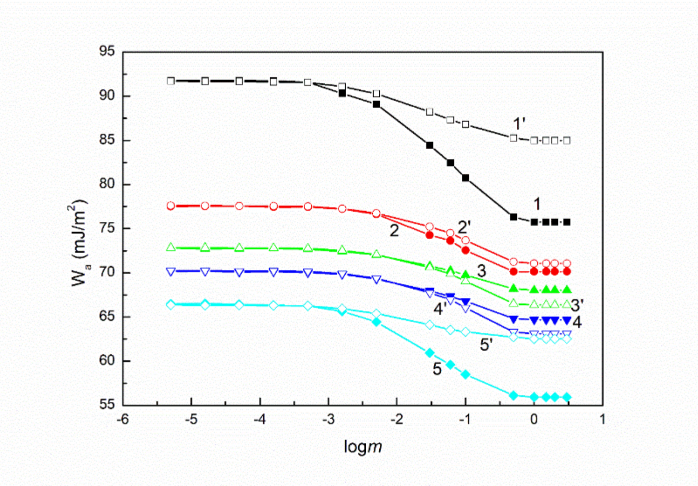

Figure S6. W_a for BPA.DA+NVP, 5CEL, 10CEL, 15CEL, 20CEL (curves 1 – 5) vs log$m$ of SE. Curves 1 – 5 correspond to the values calculated from Eq. (2), curves 1′ – 5′ to calculated from $2\left(\sqrt{\gamma_{SV}^{LW}\gamma_{LV}^{LW}}+\sqrt{\gamma_{SV}^{+}\gamma_{LV}^{-}}+\sqrt{\gamma_{SV}^{-}\gamma_{LV}^{+}}\right)=W_a$.

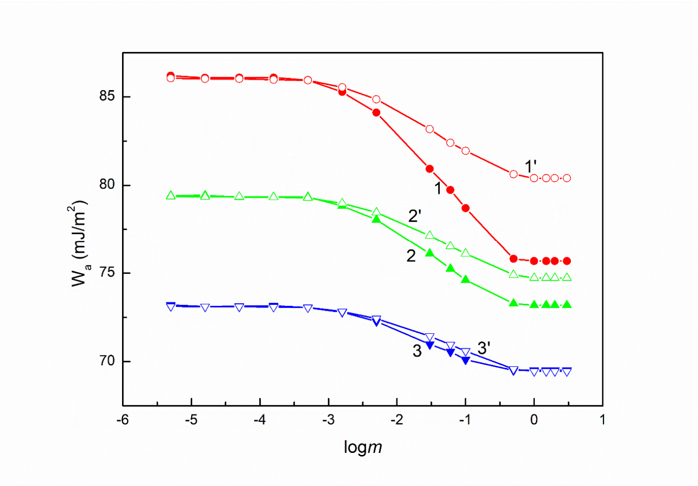

Figure S7. W_a for 5CHI, 10CHI and 15CHI (curves 1 – 3) vs log$m$ of SE. Curves 1 – 3 correspond to the values calculated from Eq. (2), curves 1′ – 3′ to calculated from $2\left(\sqrt{\gamma_{SV}^{LW}\gamma_{LV}^{LW}}+\sqrt{\gamma_{SV}^{+}\gamma_{LV}^{-}}+\sqrt{\gamma_{SV}^{-}\gamma_{LV}^{+}}\right)=W_a$.

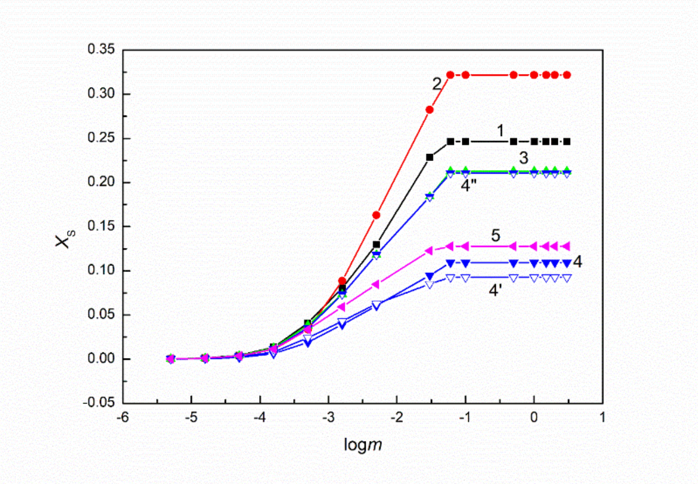

Figure S8. X_S at the solid-air interface calculated from Eq. (16) for PMMA (curve 1), quartz (curve 2), BPA.DA+NVP (curve 3), 5CEL, 15CEL, 20CEL (curves 4, 4′ and 4″) and 5CHI (curve 5) vs log*m* of SE.

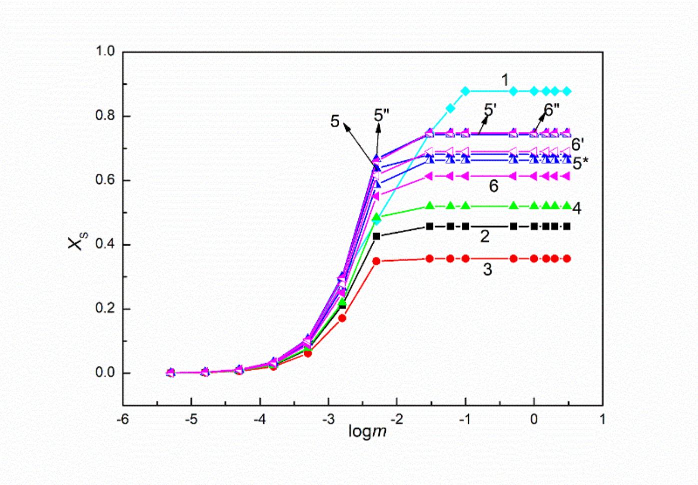

Figure S9. X_S at the solid-solution interface calculated from Eq. (16) for PTFE (curve 1), PMMA (curve 2), quartz (curve 3), BPA.DA+NVP (curve 4), 5CEL, 15CEL, 20CEL (curves 5, 5′, 5″ and 5*), 5CHI, 10CHI and 15CHI (curves 6, 6′ and 6″) vs log*m* of SE.

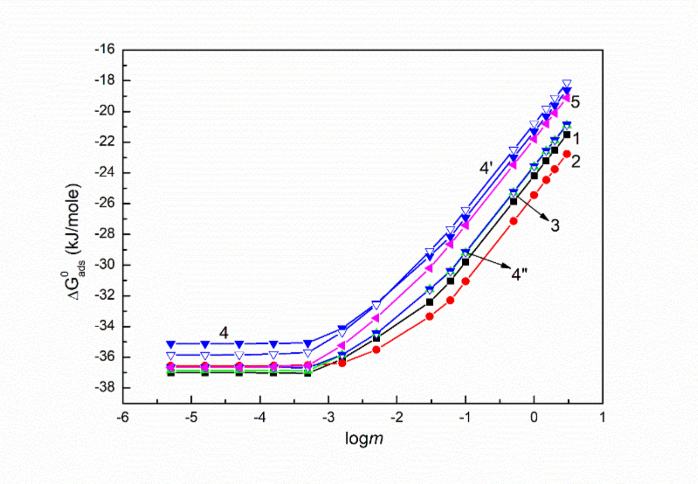

Figure S10. ΔG^0_{ads} at the solid-air interface calculated from Eq. (17) for PMMA (curve 1), quartz (curve 2), BPA.DA+NVP (curve 3), 5CEL, 15CEL, 20CEL (curves 4, 4′ and 4″) and 5CHI (curve 5) vs log*m* of SE.

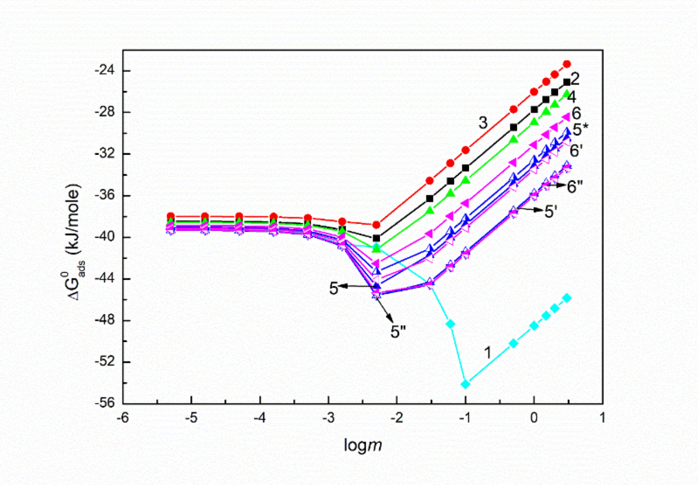

Figure S11. ΔG^0_{ads} at the solid-liquid interface calculated from Eq. (17) for PTFE (curve 1), PMMA (curve 2), quartz (curve 3), BPA.DA+NVP (curve 4), 5CEL, 15CEL, 20CEL (curves 5, 5′, 5″ and 5*), 5CHI, 10CHI and 15CHI (curves 6, 6′ and 6″) vs log*m* of SE.

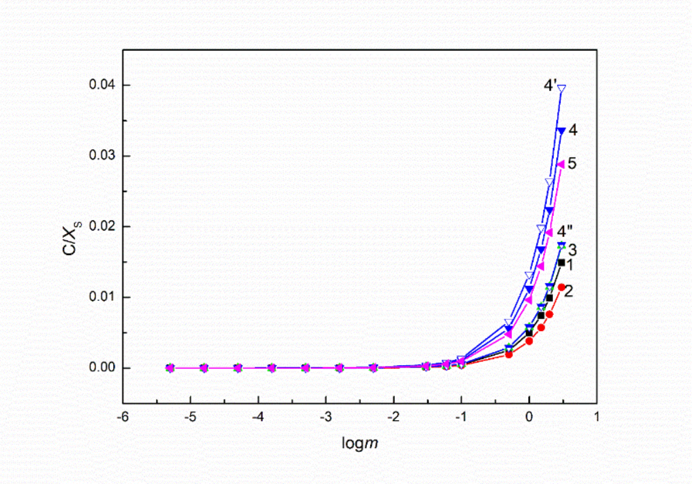

Figure S12. $\frac{C}{X_S}$ at the solid-air interface for PMMA (curve 1), quartz (curve 2), BPA.DA+NVP (curve 3), 5CEL, 15CEL, 20CEL (curves 4, 4′ and 4″) and 5CHI (curve 5) vs log$m$ of SE.

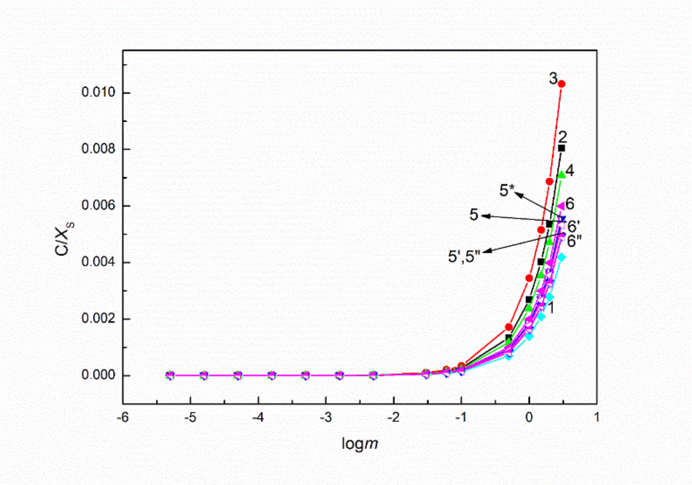

Figure S13. $\frac{C}{X_S}$ at the solid-liquid interface for PTFE (curve 1), PMMA (curve 2), quartz (curve 3), BPA.DA+NVP (curve 4), 5CEL, 15CEL, 20CEL (curves 5, 5′, 5″ and 5*), 5CHI, 10CHI and 15CHI (curves 6, 6′ and 6″) vs log$m$ of SE.
